# Identification of *Schistosoma mansoni* Infection in a Nonhuman Primate from St. Kitts More than 50 Years after Interruption of Human Transmission

**DOI:** 10.4269/ajtmh.20-0282

**Published:** 2020-09-28

**Authors:** Jennifer K. Ketzis, Manigandan Lejeune, Ian Branford, Amy Beierschmitt, Arve Lee Willingham

**Affiliations:** 1 One Health Center for Zoonoses and Tropical Veterinary Medicine, Ross University School of Veterinary Medicine, Basseterre, St. Kitts and Nevis;; 2 Department of Population Medicine and Diagnostic Sciences, Animal Health Diagnostic Center, College of Veterinary Medicine, Cornell University, Ithaca, New York;; 3 Behavioural Science Foundation, Estridge Estate, St. Kitts and Nevis

## Abstract

Transmission of *Schistosoma mansoni* was interrupted on St. Kitts, a Caribbean island, in the 1950s. With no reported cases since that time and most *Biomphalaria* spp. snail populations eliminated based on surveys in the 1970s, *S. mansoni* has been considered eliminated on St. Kitts. In 2019, *S. mansoni* eggs were found in an African green monkey (*Chlorocebus aethiops sabaeus*) that originated from St. Kitts. Nonhuman primate (NHP) infections have been considered incidental to human infections, with infections in NHPs resolving with the elimination of *S. mansoni* in the human population. An NHP with *S. mansoni* infection suggests that the NHP may be able to maintain a reservoir sylvatic cycle. Alternatively, *S. mansoni* transmission was not eliminated or *S. mansoni* has been reintroduced to St. Kitts. The occurrence of an infected NHP from St. Kitts supports the need for continuous monitoring in areas where *S. mansoni* is considered eliminated.

Schistosomiasis, a neglected tropical disease, is caused by blood trematodes of the genus *Schistosoma*. These trematodes have an indirect life cycle with aquatic snails serving as intermediate hosts and people as the final host. *Schistosoma mansoni*, the predominate species that causes intestinal schistosomiasis and uses snails of the genus *Biomphalaria* as the intermediate host, primarily occurs in Africa with introductions via the slave trade to areas of South America and the Caribbean.^1^ Efforts to interrupt transmission and eliminate the parasite are going on in endemic areas with mass drug administration of praziquantel, improvements in water systems, and molluscicide treatments or use of competitive snail species to decrease the *Biomphalaria* spp. populations.^1–4^

On St. Kitts, in the West Indies, *S. mansoni* was a significant infectious disease with approximately 25% of the population infected in the 1930s.^5,6^ Prevalence was highest in the villages of West Farm, Boyds, Old Road, and Cayon, all located near permanent and semipermanent rivers with sources in the mountainous rain forest in the center of the island (Figure 1).^5–7^ In the 1950s, there was natural abatement of *S. mansoni* infections through changes in water supply, diversion or containment of the rivers through a piping system, and snail elimination campaigns.^5^ In the 1970s, there were no human cases, and examined snail populations, which were limited to Fountain River, were not infected.^5,7,8^ Therefore, *S. mansoni* transmission was considered interrupted on St. Kitts in the 1950s and eliminated in the 1970s.

**Figure 1.**
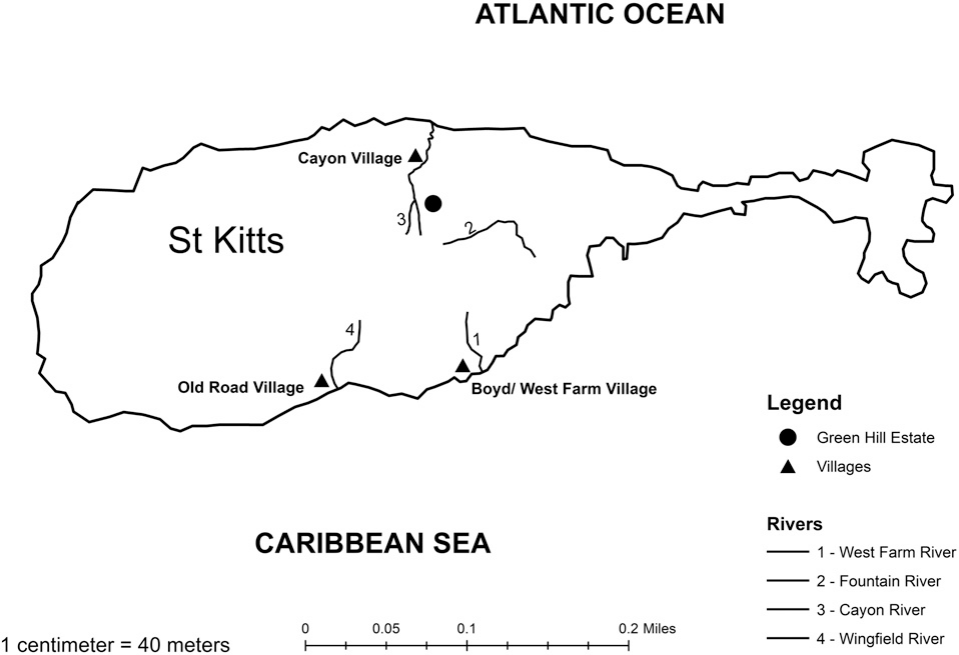
Map of St. Kitts indicating villages where schistosomiasis was prevalent in the 1930s and the relevant permanent or semipermanent rivers in the 1930s leading to these villages; the Fountain River, the location of *Biomphalaria* spp. snails in the 1970s; and Green Hill Estate, the trapping location of the positive African green monkey (*Chlorocebus aethiops sabaeus*) in 2019. All, but Wingfield, rivers leading to Old Town are now tapped near the source at > 300 m above sea level. However, the ghauts continue to have water during heavy rainfall periods.

On Caribbean islands and in localities such as Antigua, Dominican Republic, Guadeloupe, Martinique, Montserrat, Puerto Rico and Saint Lucia, where transmission is considered interrupted, there are efforts to confirm that *S. mansoni* has been eliminated.^1–3,6,9,10^ Given St. Kitts’s status of already eliminated, it has not been included in these programs, and there are no ongoing surveillance programs. However, human stool specimens are examined using direct smear or formalin sedimentation in suspect helminth infections (e.g., *Trichuris trichiura*) and for food handler annual health certificates. No *S. mansoni* infections have been reported in these examinations (personal communication with diagnostic technicians at the Joseph N. France General Hospital and Avalon Labs based on records from approximately the last 8 years, the primary public and private laboratories on St. Kitts).

Although people are the definitive final host for *S. mansoni* in the Caribbean, rodents and nonhuman primates (NHPs) can be infected.^8,11–13^ There is some uncertainty regarding the role of rodents, specifically rats, in perpetuating a sylvatic cycle; however, infection in NHPs has been considered secondary to human infections, with elimination in the human population resulting in elimination in the NHP population.^5,8^ On St. Kitts, African green monkeys (*C. a. sabaeus*) were introduced over 350 years ago, and populations also exist on Barbados, St. Maarten (St. Martin), and Tortola.^14^ For many years, interactions with people and the NHP population on St. Kitts were limited, with the NHPs predominately located in the mountainous region of the island and adventuring to coastal areas to predate on food crops.^14,15^ In 1928, when *S. mansoni* was prevalent on St. Kitts, five of seven NHPs examined were positive.^10^ In the 1960s, approximately 50 NHPs from St. Kitts were examined for *S. mansoni*, with all being negative.^16^ The lack of positive NHPs was attributed to the natural abatement of *S. mansoni* on St. Kitts and the need for human transmission to maintain infection in NHPs.

Over the last 30 years, the NHP population has increased in size and their interactions with people have increased.^14,15^ Their range is no longer limited to the mountains with NHP troops now resident in many villages on St. Kitts. Nonhuman primates are considered a nuisance species by many, and up to 60% of food crop destruction is attributed to them. Troops that have taken up residence in farming areas can be trapped and relocated, euthanized, or transferred to one of the research facilities on the island. Those transferred to one of the research facilities are tested frequently for endoparasites. Tests ranging from double centrifugation with Sheather’s sugar flotation solution, zinc sulfate with/without staining for protozoa, direct smear, to sedimentation are conducted on St. Kitts and during quarantine when the NHPs are transported to other countries. No *S. mansoni* eggs have been detected in NHP feces (personal communication with the diagnostic laboratory on St. Kitts and unpublished data of the authors). In 2015, 94 fecal samples from recently trapped NHPs were submitted to the Natural History Museum in London (a WHO Collaborating Centre for the identification and characterization of schistosome strains and their snail intermediate hosts) for PCR testing. All samples were negative for the presence of *S. mansoni* eggs (unpublished data; feces supplied by and results communicated to J. Ketzis).

In 2019, 11 NHPs were trapped from the Green Hill Estate area (Figure 1), maintained in quarantine on St. Kitts for 4 months, and then transferred to quarantine in the United States, before dispersal to research facilities. As part of routine clinical examinations, feces from all NHPs were analyzed via fecal flotation with a focus on *T. trichiura* and protozoal infections while in quarantine on St. Kitts and treated accordingly. While in quarantine in the United States, also as a part of routine examinations, a direct smear was performed by a reference laboratory, and *S. mansoni* eggs were suspected in the feces of one NHP. No photographic documentation of the eggs was obtained, and no eggs could be found in subsequent subsamples of the fecal sample. The NHP was immediately treated, and feces at treatment time were collected and frozen for PCR and further analysis.

On arrival at the laboratory for PCR analysis, a decision was made to perform qualitative fecal sedimentation before DNA extraction with the feces. Three *S. mansoni* eggs were seen (representative one in Figure 2) in a subsample (50 µL) of the sediment (approximately 1 mL) and retrieved for DNA extraction and PCR analysis. DNA extraction was performed using the Qiagen DNeasy kit (Qiagen, Hilden, Germany). PCR was performed using a published protocol to amplify a 396-bp region of cox1 gene using the primers ASMIT1 and ASMIT2.^17^ Despite retrieval of eggs for DNA extraction, the PCR assay failed to amplify the intended region for *S. mansoni*. Reasons for this are unclear and require further investigation to determine if this method, used successfully for human diagnosis, can be effectively used in the NHP. Nevertheless, the unique morphology of eggs is confirmatory for *S. mansoni* diagnosis.

**Figure 2.**
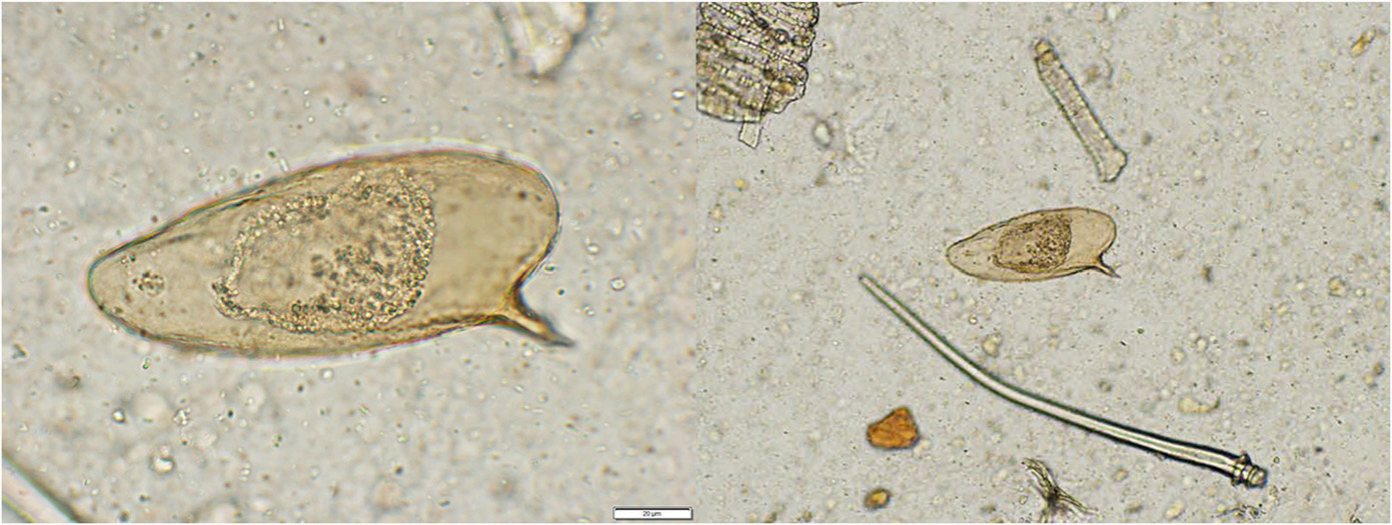
*Schistosoma mansoni* eggs isolated from the feces of the African green monkey (*Chlorocebus aethiops sabaeus*) in 2019.

This finding of *S. mansoni* eggs in the feces of an NHP from St. Kitts is the first documentation of *S. mansoni* infection on St. Kitts since the 1950s. Although the NHP was no longer on St. Kitts at the time the infection was detected, given the quarantine facilities, use of potable water, and the management of the NHP, the only source of infection could have been while the NHP was resident on St. Kitts. The negative samples before shipment are likely due to the choice of a flotation method instead of the preferred sedimentation method for *S. mansoni* being used; however, even when sedimentation or direct smears are used, detection levels can be low.^18,19^

The origin of this infection is unknown. Potentially, *S. mansoni* has been recently reintroduced via the immigration or visitation of people from endemic Caribbean localities. Much of the Caribbean population is fluid with family members across islands traveling easily between them. Alternatively, *S. mansoni* transmission was either not eliminated or only below detectable limits and is still present in the human population. Another possibility is that *S. mansoni* has continued in a sylvatic cycle. This is in contradiction to what has been proposed regarding the NHP as final hosts.^8^ However, Green Hill Estate, the area from which this NHP was trapped, is in the vicinity of the head of the Cayon River, one of the previous sources of this parasite. Although the last testing and documentation of *Biomphalaria* spp. at the head of the Cayon River in 1958 were negative for *S. mansoni* and the only reported remaining population in the 1977 survey of *Biomphalaria* spp. was at the Fountain River, the *Biomphalaria* spp. population could have recovered at Cayon.

The finding of *S. mansoni* in the NHP raises important issues. First, the role of NHPs in perpetuating *S. mansoni* in a region might require reexamination, especially if elimination programs are to be effective. Second, this finding emphasizes the need for continued surveillance and monitoring of snail populations, even in areas where transmission has been stopped and elimination has been assumed.

Based on this report of *S. mansoni*, quarantined NHPs are being treated with praziquantel and parasite screening now includes sedimentation. In addition, snail surveys on St. Kitts are planned for the latter half of 2020 as well as serum (for antibody testing) and fecal collections (for egg identification) from NHPs trapped from Green Hill Estate and the Fountain River and Cayon River areas. Last, the St. Kitts and Nevis Medical Association and diagnostic laboratories on St. Kitts will be informed of this finding, although the risk of human infection and transmission is still likely low. In conclusion, the finding of this infection, in an NHP after over 50 years of transmission elimination in people in the locality, supports monitoring to ensure transmission does not reoccur if elimination of the intermediate host snail population has not been fully achieved.
